# Synaptic phospholipids as a new target for cortical hyperexcitability and E/I balance in psychiatric disorders

**DOI:** 10.1038/s41380-018-0053-1

**Published:** 2018-05-09

**Authors:** Carine Thalman, Guilherme Horta, Lianyong Qiao, Heiko Endle, Irmgard Tegeder, Hong Cheng, Gregor Laube, Torfi Sigurdsson, Maria Jelena Hauser, Stefan Tenzer, Ute Distler, Junken Aoki, Andrew J. Morris, Gerd Geisslinger, Jochen Röper, Sergei Kirischuk, Heiko J. Luhmann, Konstantin Radyushkin, Robert Nitsch, Johannes Vogt

**Affiliations:** 1 Department of Neurology, University Medical Center, Johannes Gutenberg-University, Mainz, Germany; 2 Institute for Microscopic Anatomy and Neurobiology, University Medical Center, Johannes Gutenberg-University, Mainz, Germany; 3 Institute of Clinical Pharmacology, Goethe University Frankfurt, Frankfurt, Germany; 4 Institute for Integrative Neuroanatomy, Charité – Universitätsmedizin, Berlin, Germany; 5 Institute of Neurophysiology, Neuroscience Center, Goethe University Frankfurt, Frankfurt, Germany; 6 Institute for Immunology, University Medical Center, Johannes Gutenberg-University, Mainz, Germany; 7 Focus Program Translational Neuroscience, Johannes Gutenberg-University, Mainz, Germany; 8 Graduate School of Pharmaceutical Sciences, Tohoku University, Aoba-ku, Sendai Japan; 9 Division of Cardiovascular Medicine, Gill Heart Institute, University of Kentucky, Lexington, KY USA; 10 Institute of Physiology, University Medical Center, Johannes Gutenberg-University, Mainz, Germany; 11 Institute for Translational Neuroscience, University Medical Center, Westfälische Wilhems-University Münster, Albert-Schweitzer-Campus, Münster, Germany

**Keywords:** Neuroscience, Schizophrenia

## Abstract

Lysophosphatidic acid (LPA) is a synaptic phospholipid, which regulates cortical excitation/inhibition (E/I) balance and controls sensory information processing in mice and man. Altered synaptic LPA signaling was shown to be associated with psychiatric disorders. Here, we show that the LPA-synthesizing enzyme autotaxin (ATX) is expressed in the astrocytic compartment of excitatory synapses and modulates glutamatergic transmission. In astrocytes, ATX is sorted toward fine astrocytic processes and transported to excitatory but not inhibitory synapses. This ATX sorting, as well as the enzymatic activity of astrocyte-derived ATX are dynamically regulated by neuronal activity via astrocytic glutamate receptors. Pharmacological and genetic ATX inhibition both rescued schizophrenia-related hyperexcitability syndromes caused by altered bioactive lipid signaling in two genetic mouse models for psychiatric disorders. Interestingly, ATX inhibition did not affect naive animals. However, as our data suggested that pharmacological ATX inhibition is a general method to reverse cortical excitability, we applied ATX inhibition in a ketamine model of schizophrenia and rescued thereby the electrophysiological and behavioral schizophrenia-like phenotype. Our data show that astrocytic ATX is a novel modulator of glutamatergic transmission and that targeting ATX might be a versatile strategy for a novel drug therapy to treat cortical hyperexcitability in psychiatric disorders.

## Introduction

Synaptic transmission is a fundamental requirement for normal brain function [1]. Alterations of transmission at excitatory synapses are involved in a variety of neuropsychiatric disorders such as schizophrenia [2, 3]. Proper synaptic transmission relies on the regulation of neurotransmitters by different processes and is essential for neuronal communication. Recent research showed that bioactive phospholipids play an important role in synaptic neurotransmission and plasticity. Specifically, lysophosphatidic acid (LPA) was shown to affect synaptic strength and plasticity of central synapses [4–6]. Synaptic LPA is modulated by the plasticity-related gene 1 (PRG-1) [7], which is expressed at the postsynaptic density of excitatory synapses on cortical and hippocampal glutamatergic neurons and plays an important role in maintaining synaptic homeostasis [4, 6, 8]. Loss of PRG-1-mediated LPA regulation leads to cortical network hyperexcitability due to a higher presynaptic glutamate release probability, which is mediated by increased synaptic LPA levels acting on presynaptic LPA-2 receptors [4]. Recently, a human mutation (PRG-1^R345T^) leading to a loss-of-PRG-1 function and a dysregulation of synaptic LPA signaling was shown to lead to cortical hyperexcitability and an altered sensorimotor gating (pre-pulse inhibition [PPI] in mice and an altered P50 wave in humans), an endophenotype of psychiatric disorders in mice and man [5]. The role of altered synaptic lipid signaling in psychiatric disorders is supported by recent data showing that PRG-1-deficiency results in hypermotility symptoms [9] and is in line with the glutamatergic hypothesis of an altered cortical excitation/inhibition (E/I) balance in the pathophysiology of psychiatric disorders [10–12]. Extracellular LPA is synthesized by autotaxin (ATX), a lyso phospholipase D (PLD) able to hydrolyze lysophosphatidyl choline (LPC) to LPA. LPC is transported across the blood–brain barrier in an Mfsd2a-dependent manner [13] and is present in the brain in low concentrations. Thus, the ATX-dependent LPC to LPA conversion is a rate-limiting step for phospholipid signaling in the brain. Moreover, LPA is rapidly degraded by lipid phosphate phosphatases, which are present in the synaptic compartment [14]. Therefore, as a remote LPA synthesis is not able to produce synaptic LPA levels necessary for the modulation of glutamatergic transmission, we aimed to find the source of synaptic LPA. To this end, we detected the LPA-synthesizing enzyme ATX in astrocytic perisynaptic lamellae engulfing excitatory synapses. As pharmacological inhibition of ATX was effective in decreasing LPA levels in the cerebrospinal fluid (CSF) [5], we speculated that inhibiting ATX might be a versatile strategy in treating pathophysiological states associated with cortical hyperexcitability. Our data revealed that ATX release from astrocytes was regulated by glutamate and was dependent on astrocytic glutamate receptors. Using pharmacological ATX inhibition and cell type-specific ATX deletion in astrocytes, we found a reduction in the frequency of miniature and spontaneous excitatory postsynaptic currents (mEPSCs and spEPSCs) to wild-type levels under conditions of hyperexcitability. Moreover, electrophysiological measurements in freely moving PRG-1^-/-^ mice revealed that ATX inhibition normalized altered gamma coherence of cortico–cortical connections, an endophenotype of psychiatric disorders [15]. Using a mouse model expressing a human PRG-1 loss-of-function single-nucleotide polymorphism (SNP; PRG-1^R346T^), which leads to a dysregulated cortical information processing, and a well-established ketamine mouse model of schizophrenia [16], we could demonstrate the efficacy of pharmacological ATX inhibition in reducing hyperexcitatory cortical states and the associated characteristic schizophrenia-like phenotypes.

## Materials and methods

Mice astrocytic cultures were prepared from P0-P1 mice cortices according to standard procedures. Briefly, after removing meninges, cortices were dissociated in Trypsin-EDTA (Sigma Aldrich, Germany), centrifuged and re-suspended in Dulbecco’s modified Eagle’s medium (DMEM) plus 10% fetal bovine serum (FBS), 100 U/ml penicillin, 100 µg/ml streptomycine (Gibco, Germany) and 2mM l-glutamine (Stem cell technologies, Germany). Cells were then seeded in poly-l-lysine (10 µg/µl, Sigma Aldrich, Germany) pre-coated cell culture plates (Greiner Bio-One, Germany) and maintained in a humidified incubator (5% CO_2_). After 3 h of culture, cells were shaken and medium was renewed. After 7 days in vitro (DIV), cells were shaken again for removal of possible microglia.

### Immunohistochemistry

Brain slices were incubated with antibodies against ATX (4F1 1:1000) [8, 17], glial fibrillary acidic protein (GFAP, 1:1500), Ezrin (3C12 1:1000, Sigma Aldrich, Germany), vesicular glutamate transporter 1 (VGluT1) and vesicular GABA transporter (VGAT) (both 1:1000, Synaptic Systems, Germany) as described [8]. Electron microscopic studies were performed as described [4]. For 3,3’-diaminobenzidine (DAB) conversion, biotinylated secondary antibodies were used and DAB staining was performed as described earlier [4, 18].

### Stimulation of astrocytes

After 8–9 DIV, astrocytes were cultivated for 2–4 h in DMEM without FBS, glutamine or Pen/Strep. The cells were then stimulated with 500 µM glutamate for 15 min and incubated with fresh DMEM (for western blotting [WB]) or supplemented with 100 µM C17-LPC (Avanti, USA) for assessment of ATX activity via mass spectrometry. In control (i.e., non-stimulated) wells from the same preparation, only media change were performed. Inhibitors (DL-TBOA [Tocris, United Kingdom], APV [BioTrend, Germany], DNOX, LY367385 [Tocris, Germany]) were applied 10 min before glutamate stimulation. After stimulation, supernatant and cells were collected at different time points, frozen in liquid nitrogen and stored at –80 °C until further processing.

### Western blotting

Total protein from astrocytes cell culture supernatant was extracted and concentrated using Vivaspin (Sigma Aldrich, Germany) centrifugal concentrator according to the producer’s advice. WB was performed according to standard procedures as described [19].

### Mass spectrometry and confocal live imaging

In all, 100 µl of supernatant was collected from each sample and directly frozen. After washing, cells were collected, pelleted, re-suspended in 50 µl of phosphate-buffered saline, and frozen at –80 °C. C17-LPA levels of the samples were measured via mass spectrometry as described [8].

Confocal live imaging of cultivated astrocytes was performed after transfection with 3 µg of ATX-green-fluorescent protein (GFP)-myc plasmid with Lipofectamine 2000 (Thermo Fisher Scientific, USA). Six to 8 days after transfection, cells were cultivated for 2 h in non-supplemented DMEM and imaged at 30 °C using a top-stage incubator with an inverse confocal Leica SP5 microscope. After baseline imaging, control and stimulated astrocytes were imaged at the indicated time points where pictures were taken every 30 s for 5 min. Gray values of ATX-GFP vesicles in the processes were quantified using ImageJ and a predefined unbiased threshold (Huang, ImageJ, Bethesda, MD, USA), which allowed detection of single vesicles.

### Mouse lines

PRG-1^-/-^ and ATX^fl/fl^ mice were generated and genotyped as described [4, 20]. Astrocycte-specific ATX deletion was obtained by breeding ATX^fl/fl^ mice with a mouse line expressing Cre under the GFAP promotor [21]. Transgenic mice expressing the human PRG-1 variant PRG-1^R346T^ were obtained using CRISPR/Cas9 technology to induce a point mutation. Hereby a construct was designed to induce a site mutation resulting in a change from arginine (R; AGG) to threonine (T; ACG) at position 346 (345 in humans). After mouse line generation, the PRG-1^R346T^ mutation was confirmed by sequencing and off-target search was successfully performed. By introduction of the site mutation, a restriction site for BmgBI (5ʹ-CACGTC-3ʹ) was generated, which was used for further genotyping. All experiments were conducted in accordance with the national laws for the use of animals in research and with the European Communities Council Directive 86/609/EEC, and approved by the local ethical committee (Landesuntersuchungsamt Rheinland-Pfalz G 12-1-096). Experiments were designed to minimize the number of animals used.

### Electrophysiological measurements

All experiments were performed on CA1 neurons in acute slices from 16 to 19 days old or from 6- to 8-week-old C57BL/6 mice or on indicated transgenic mice raised on a C57BL/6 background. The transgenic mouse lines used in this study are described elsewhere [4, 20]. For field recordings, slices were kept for at least 1 h at room temperature in an oxygenated ACSF field and pre-warmed for 30 min at 32.5 °C. Slices non-responsive to ketamine application were excluded. ATX inhibition was performed using HA130 [5, 22]. Spontaneous, evoked and miniature excitatory currents (spEPSCs, eEPSCs and mEPSCs) were acquired with an ELC-03XS amplifier (NPI electronics, Germany) and using spike 2 software (CED products, Germany). The signals were filtered at 2 kHz and sampled at a rate of 10 kHz. Recordings were performed with GB200F-10 filament microelectrode (3–6 MOhm) in cells, voltage-clamped at –70 mV. spEPSCs and mEPSCs were pharmacologically isolated with Gabazine (10 µM) and APV (50 µM) or TTX (0,5 µM) and Gabazine, respectively. Paired-pulse ratio (PPR) were recorded in CA1 pyramidal cells by stimulating Schaeffer Collateral fibers with a bipolar Tungsten electrode at 50 ms inter stimulus interval. Duration of the pulse was set to 200 µs with the pulse intensity between 10 and 30 µA. eEPSCs were recorded in presence of APV, Gabazine and CGP55845 to block NMDA-, GABAA- and GABAB-receptors, respectively. PPR was calculated by dividing the mean amplitude of the second eEPSC by the mean amplitude of the first eEPSC (average of 20 trials). For ATX inhibition with 1 µM PF8380, slices were incubated for 1 h before whole-cell configuration as described [5, 23].

In vivo electrophysiological recordings were performed as described [24] and were approved by the local authorities. Briefly, anesthetized animals were chronically implanted with electrodes (WE3PT10.5F3, MicroProbes, Gaithersburg, MD, USA) in the medial entorhinal cortex (MEC; 3.1 mm, lateral to the midline, 0.2 mm anterior to the transverse sinus at an angle of 6° in the anterior-to-posterior direction in the sagittal plane) and in the hippocampal CA1-region (1.5 mm lateral to the midline, 1.5 mm anterior-to-posterior and 1.2 mm dorsal-to-ventral at an angle of 0°). Neural data were acquired using a 16-channel head stage and a Digital Lynx data acquisition system (Neuralynx, USA); the animal’s position was monitored using two light-emitting diodes mounted on the head stage. To extract local field potentials (LFPs), the neural signals were bandpass filtered between 1 and 1000 Hz and digitized at 2 kHz. Recordings were repeated for 2 days. Recording location was verified in cresyl violet stained sagittal brain slices.

### Field potential analysis

Coherence of neuronal activity between the MEC and in the CA1 was computed using the multitaper method (MATLAB routines provided by K. Harris, University College London, London, UK, and G. Buzsáki, New York University, New York, NY, USA). Field potentials were divided into 500 ms segments; coherence was then computed by averaging the cross-spectral densities of two field potential signals across data windows and tapers and normalizing to the power spectral densities of each signal. To measure oscillatory power, the LFP signals were convolved with a series of Morlet wavelets with center frequencies ranging from 1 to 100 Hz and a length of three cycles. Power was calculated by taking the absolute value of the wavelet transform and averaging across time to obtain the power spectrum. For statistical analysis, coherence and power were averaged within the theta (7–12 Hz) and gamma (30–70 Hz) frequency ranges.

### Behavior

For open field assessment, mice were placed in an arena (40 × 40 cm) with video tracking and were allowed to freely explore the chamber. Evaluation of PPI was performed as described [5]. Briefly, PPI was determined using a 120 dB/40 ms startle pulse applied alone or preceded by a pre-pulse stimulus of 70, 75 or 80 dB intensity. Amplitudes of the startle response were averaged and PPI was calculated as the percentage of the startle response using the following formula:

$${\mathrm{PPI}}\left( {\mathrm{\% }} \right) \\ 	= 100 - \left( {\frac{{startle\,amplitude\,after\,pre - pulse\,\& \,pulse}}{{startle\,amplitude\,after\,pulse\,only}} \times 100} \right)$$

Ketamine application for open field (16 mg/kg [16]) and for PPI (30 mg/kg [25]) assessment was performed as previously reported. For ATX inhibition, PF8380 was applied 3 h before the experiment (30 mg/kg i.p. in dimethyl sulfoxide (DMSO) [23]).

### Statistics and data analysis

All statistical analyses were performed with GraphPad Prism software (version 6). The data are expressed as the mean ± standard error of the mean (SEM). Appropriate statistical tests were chosen based on the experimental condition. Normal distribution of non-normalized data was assessed using the Kolmogorow–Smirnov test. When normal distribution was rejected, a non-parametric test was used. Significance was considered for *p* ≤ 0.05.

## Results

### ATX is expressed in perisynaptic astrocytic processes (PAPs) at the glutamatergic synapse

**Fig. 1:**
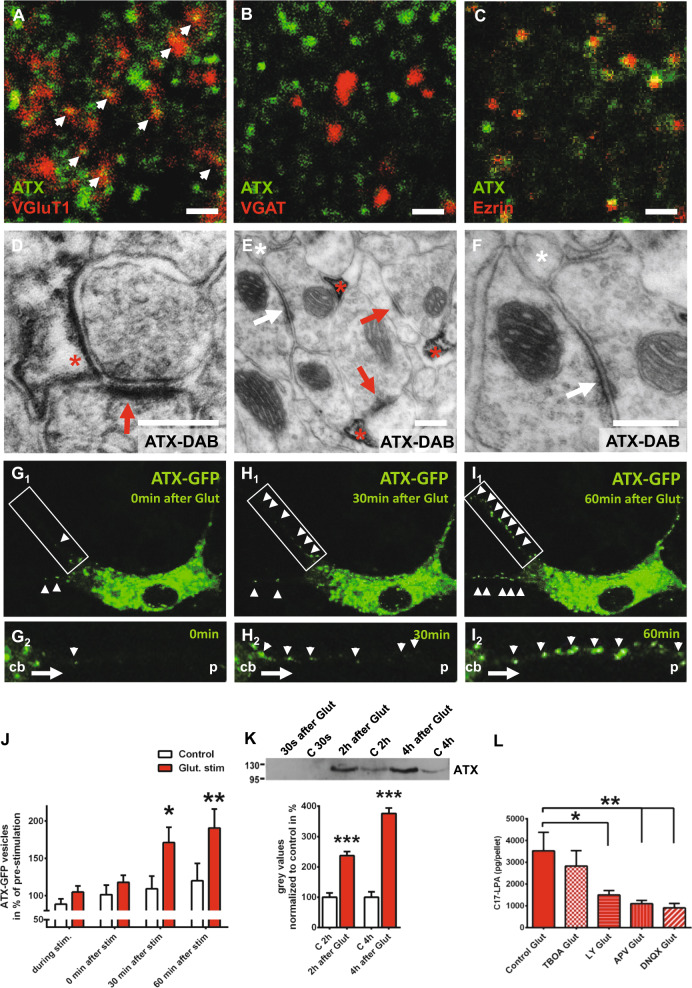
ATX is expressed by perisynaptic astrocytic processes (PAPs) at excitatory synapses and is regulated by glutamate. **a, b** ATX shows abundant colocalization with VGluT1, a marker of excitatory synapses, but not with VGAT, a marker of inhibitory synapses. **c** ATX is expressed at PAPs as shown by colocalization with Ezrin, a specific marker for PAPs. Scale bars a–c: 1 µm. **d** Electron microscopic image showing ATX expression (displayed by DAB-precipitation [red asterisk] at the astrocytic membrane close to the synapse) on PAP membranes surrounding glutamatergic synapses. Dense membrane in the postsynaptic compartment (red arrow) represents the postsynaptic density (PSD) of glutamatergic synapses (see also Fig. S1B). **e** Immune electron microscopic analysis revealed no ATX expression in astrocytic processes (white asterisk) at inhibitory synapses (white arrow). In contrast, ATX expression (shown by DAB precipitation) was prominent in astrocytic processes (red asterisk) at excitatory synapses (red arrow, see also Fig. S1C,D). **f** Higher magnification showing the inhibitory synapse (white arrow) and the adjacent ATX-negative astrocytic process (white asterisk). Scale bars d–f: 200 nm. **G**_**1**_**-I**_**1**_ Live imaging of astrocytes transfected with an ATX-GFP-expressing construct shows a clear increase of ATX-GFP-positive vesicles transported along their processes towards the periphery after glutamate stimulation (500 µM for 15 min). **G**_**2**_**-I**_**2**_ Live-imaging pictures at higher magnification show transport of ATX-GFP-positive vesicles (white arrow heads) along an astrocytic process from the cell body (cb) toward the periphery (p). In contrast, ATX-GFP-positive vesicles were rarely seen in processes of control, non-stimulated astrocytes (Fig. S1E). **j** Quantitative analysis of ATX-GFP vesicles in astrocytic processes, during glutamate stimulation and at 0, 30 and 60 min after glutamate stimulation (*n* = 13 control astrocytes and 14 glutamate-stimulated astrocytes; two-way RM ANOVA with Bonferroni post hoc). **k** ATX secretion in the astrocytic culture supernatant was significantly increased upon glutamate stimulation (500 µM Glut stimulation for 15 min) when compared with supernatant from control (c), non-stimulated astrocytic cultures as shown by western blot (*n* = 5 for 2 h values and *n* = 4 for 4 h values, two-tailed* t*-test). **l** C17-LPA synthesis in the astrocytic compartment was significantly decreased upon application of specific glutamate receptor inhibitors (50 µM LY367385 for mGluR1a, 50 µM APV for NMDA-Rs and 10 µM DNQX for AMPA-Rs), whereas 40 µM DL-TBOA, an inhibitor of astrocytic glutamate transporters, did not affect astrocytic C17-LPA levels as shown by mass spectrometry analyses (*n* = 6 experiments per group, one-way RM ANOVA with Bonferroni post hoc). Bars represent mean ± SEM. *p < 0.05, **p < 0.01, ***p < 0.001

Using colocalization studies with markers of glutamatergic (VGluT1) and GABAergic (VGAT) synapses in the adult brain, we detected abundant ATX expression at excitatory synapses (Fig. 1a) but no ATX expression at inhibitory synapses (Fig. 1b). Further analyses using high-sensitivity immunofluorescence revealed a punctae-like ATX immunosignal and occasional labeling of astrocytic processes defined by glial fibrillary acidic protein GFAP staining (Fig. S1A), while labeling of astrocytic cell bodies was hardly present, as previously described [26]. Colocalization with ezrin, a marker of PAPs [27], suggested synaptic ATX expression in dendritic regions (Fig. 1c). Finally, electron microscopic analysis using DAB precipitation clearly confirmed ATX expression in the astrocytic processes surrounding glutamatergic synapses, which are defined by their asymmetric postsynaptic densities (Fig. 1d and Fig. S1B) [28]. Interestingly, electron microscopic analyses confirmed abundant ATX expression in astrocytic processes at excitatory synapses but not at inhibitory synapses in the hippocampal CA1-region (Figs. 1e, f), as well as in the cortex, as shown in the layer 4 of the auditory cortex (Fig. S1C,D). This suggests an intracellular sorting of ATX in astrocytes toward the astrocytic processes covering excitatory synapses.

### Astrocytic ATX secretion and activity are regulated by glutamatergic signaling

In order to understand this specific ATX expression pattern, we performed live imaging of primary astrocytes expressing an ATX–GFP fusion protein. In the culture setting devoid of the neuronal compartment, astrocytes displayed only sparse ATX–GFP signals along their processes (Fig. S1E). However, after glutamate stimulation we detected a clear transport of ATX–GFP vesicles along astrocytic processes (Figs. 1g-j), which suggests a dynamic regulated intracellular ATX sorting and ATX transport along astrocytic processes toward excitatory synapses by glutamatergic signaling. As ATX is a transmembrane protein with a catalytic domain facing the extracellular space, where it is eventually shed [29], we speculated that ATX is released from astrocytic processes in a glutamate-dependent manner and exhibits its enzymatic activity converting LPC to LPA in the synaptic space. Indeed, we detected a significant increase in ATX levels in the supernatant of cultured astrocytes upon glutamate stimulation (Fig. 1k). Moreover, after adding the unnatural precursor C17-LPC we detected a clear increase in ATX´s enzymatic activity and found significantly increased levels of newly synthesized C17-LPA upon glutamate stimulation (Fig. S1F-K). Here, C17-LPA reached significantly higher levels within 3 min after glutmatergic stimulation (when compared with non-stimulated conditions, Fig. S1J). We then characterized glutamate signaling pathways involved in astrocytic ATX upregulation and inhibited astrocytic glutamate transporters (using TBOA), as well as glutamate receptors present on astrocytes (α-amino-3-hydroxyl-5-methyl-4-isoxazolepropionic acid receptor (AMPA), N-methyl-D-aspartate receptor (NMDA), mGluR1a). Although blocking glutamate transporters did not affect glutamate-induced increase of enzymatic ATX activity, inhibition of AMPA, NMDA or mGluR1a receptors (using the specific inhibitors DNQX, APV and LY367385, respectively) significantly inhibited glutamate-dependent increase of ATX activity (Fig. 1l). In sum, our data indicate that glutamate released from presynaptic terminals acts on glutamatergic receptors present on astrocytes where it induces ATX-sorting and ATX-transport toward excitatory synapses. At the synapse, ATX is released and is able to exert its enzymatic function by converting LPC to LPA in the synaptic cleft.

### ATX inhibition normalizes cortical hyperexcitability and behavior in two animal models of psychiatric disorders

**Fig. 2:**
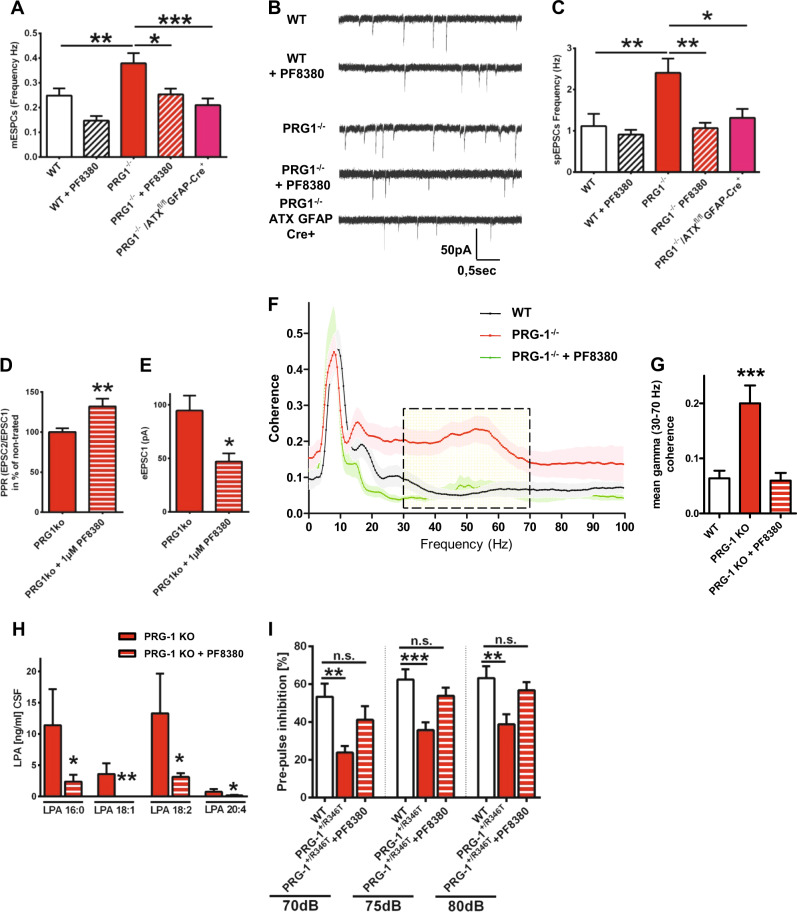
ATX inhibition rescues neuronal hyperexcitability and PPI deficits in PRG-1-deficient animals. **a** Miniature excitatory postsynaptic currents (mEPSCs) in CA1 pyramidal neurons of wild-type (WT) slices were not affected by ATX inhibition with 1 µM PF8380. However, in PRG-1^-/-^ slices, which display higher mEPSC frequency, ATX inhibition significantly diminished mEPSC frequency to WT levels. In line, astrocyte-specific ATX deletion significantly decreased mEPSC frequency in PRG-1^-/-^ animals to WT levels. Amplitudes (see Fig. S2C) were not affected supporting a presynaptic effect (*n* = 13 WT neurons, 8 PF8380-treated WT neurons, 10 PRG-1^-/-^ neurons, 10 PF8380-treated PRG-1^-/-^ neurons and 10 PRG-1^-/-^/ATX^fl/fl^:GFAP-Cre^+^ neurons; one-way ANOVA with Bonferroni post hoc). **b** Original traces showing spontaneous excitatory postsynaptic currents (spEPSCs) of neurons under different conditions. **c** Although ATX inhibition via PF8380 application did not alter spontaneous excitatory (spEPSCs) frequency in WT slices, higher spontaneous frequency observed in PRG-1^-/-^ neurons was significantly decreased to WT levels. Similarly, genetic deletion of ATX in astrocytes decreased spontaneous excitatory frequency in PRG-1^-/-^ neurons to WT levels, whereas amplitudes were not affected (see Fig. S2D) (n = 11 WT neurons, 12 PF8380-treated WT neurons, 19 PRG-1^-/-^ neurons, 17 PF8380-treated PRG-1^-/-^ neurons and 18 PRG-1^-/-^/ATX^fl/fl^:GFAP-Cre^+^ neurons; Kruskal–Wallis test with Dunn’s multiple comparisons post hoc test). **d** Paired-pulse ratio (PPR) in PRG-1^-/-^ animals was significantly increased after ATX inhibition. **e** ATX inhibition by PF8380 decreased the first eEPSC(1) in PRG-1^-/-^ animals. **f** Coherence analysis of simultaneous recordings of field potentials in layer II/III of the medial entorhinal cortex (MEC) and the hippocampal CA1 in freely moving mice revealed significantly higher coherence in the gamma range (30–70 Hz, dashed box) in PRG-1^-/-^ mice. Interestingly, LPA reduction by application of the ATX inhibitor PF8380 reduced this coherence in PRG-1^-/-^ mice to WT level (see also Fig. S3A, D-F). **g** Quantitative analysis revealed significantly increased mean gamma coherence in PRG-1^-/-^ mice, which was reduced to WT levels upon PF8380-treatment (*n* = 15 WT mice, 12 untreated PRG1^-/-^ and 7 PF8380-treated PRG1^-/-^ mice; one-way ANOVA with Bonferroni post hoc; see also Fig S3B). **h** In order to assess the efficiency of ATX inhibition by PF8380, LPA levels were measured in the CSF after PF8380 application (30 mg/kg body weight). Here, we found a clear decrease in the main LPA species (LPA 16:0, 18:1, 18:2 and 20:4; *n* = 6 for all groups; one tailed, Mann–Whitney test; see also Fig. S2C). **i** Pre-pulse inhibition (PPI) at all tested loudness levels was significantly decreased in a mouse line expressing a monoallelic human PRG-1 single-nucleotide polymorphism (SNP). However, PF8380 application rescued decreased PPI to WT levels (*n* = 9 WT mice, 15 untreated PRG1^+/R346T^ and 15 PF8380-treated PRG1^+/R346T^ mice; one-way ANOVA with Bonferroni post hoc; see also Fig. S3G-I). Bars represent mean ± SEM. *p < 0.05, **p < 0.01, ***p < 0.001

As recently reported, increased synaptic LPA levels augmented mEPSCs frequency but not their amplitudes, pointing to an increased presynaptic release probability. However, disruption of LPA signaling at glutamatergic synapses (by deletion of the presynaptic LPA_2_ receptor) reduced mEPSCs frequency in conditions of glutamate-mediated cortical hyperexcitability in PRG-1^-/-^ animals [4, 8]. We therefore used PRG-1^-/-^ animals and inhibited ATX using PF8380 [5, 23] finding that ATX inhibition reduced both mEPSC and spontaneous (sp)EPSC frequencies to control levels (Figs. 2a-c and Fig.S2B-D). However, ATX inhibition did not alter mEPSC or spEPSC frequencies in wild-type slices (Figs. 2a-c and Fig. S2A,C,D) and had no effect on wild-type animal behavior (Fig. S2E,F). In line, cortical hyperexcitability in PRG-1^-/-^ animals was rescued by cell type-specific genetic deletion of ATX in astrocytes (using a PRG-1^-/-^/ATX^fl/fl^/GFAP-Cre mouse line, Figs. 2a-c). However, in wild-type animals, which display normal cortical excitability, astrocytic ATX deletion did not alter neuronal activity or animal behavior (Fig. S2G-L) supporting the idea of a specific effect of ATX inhibition in conditions of cortical hyperexcitability. Interestingly, in addition to normalizing cortical hyperexcitability, ATX inhibition rescued presynaptic plasticity (Fig. 2d), which is altered in PRG-1^-/-^ neurons as previously described [8]. Here, ATX inhibition decreased the first EPSC (eEPSC1) corroborating the presynaptic action of synaptic LPA and supporting the idea that higher synaptic LPA levels increase presynaptic release probability and impair thereby short-term presynaptic plasticity (Fig. 2e). In sum, these data suggest that cortical hyperexcitability is regulated by synaptic LPA and that ATX inhibition is a selective and versatile strategy to normalize glutamate-induced hyperexcitable cortical states.

To assess the effect of cortical hyperexcitability in vivo, we performed simultaneous field potential recordings of hippocampal CA1 neurons and of layer II/III entorhinal cortex neurons (which connect to hippocampal CA1 neurons) in freely moving PRG-1^-/-^ animals and in wild-type litters (Fig. S3A). Here, we found that the hyperactive cortical state present in PRG-1^-/-^ animals [4, 6, 8] resulted in a significant increase in the mean gamma coherence (Figs. 2f, g and Fig S2B), which is typically altered in patients with psychiatric disorders like schizophrenia [15, 30]. To examine the utility of ATX inhibition as a potential therapeutic option, we treated PRG-1^-/-^ animals with the ATX-inhibitor PF8380 [23]. This was highly effective at reducing LPA moieties in the CSF (Fig. 2h and Fig. S3C) and rescued mean gamma coherence to wild-type levels (Figs. 2f, g). Moreover, ATX inhibition decreased hypermotility of PRG-1^-/-^ mice in an open field setting (Fig. S3D-F), which is a typical symptom for psychiatric disorders and was reported to be mediated by loss of PRG-1-mediated regulation of synaptic LPA levels [9]. To directly prove the idea that loss of synaptic LPA regulation and subsequent increase in cortical excitability induces an endophenotype for psychiatric disorders, we analyzed transgenic animals expressing a previously described human SNP of PRG-1, which leads to a loss of PRG-1's regulatory function (PRG-1^R346T^ [5]). Here, the PRG-1^R346T^ mutation induced an altered glycosylation leading to a loss of PRG-1´s ability to take up LPA (see also Figure S3G-I). Interestingly, human carriers of this mutation (PRG-1^R345T^) displayed changes in sensory information processing (as shown by altered P50 values), which are regarded as an endophenotype for schizophrenia [5]. We found significantly reduced PPI values in PRG-1^+/R346T^ mice (Fig. 2i), which reflect a sensorimotor cortical filter function. PPI analyzes are a long-standing paradigm related to psychiatric diseases like schizophrenia for which humans and rodents are assessed in similar manner [31]. In order to test our findings on this endophenotype, we treated the PRG-1^+/R346T^ carrier mice with PF8380. Here, ATX inhibition normalized the reduced PPI startle responses to wild-type levels (Fig. 2i) and corroborated the idea of ATX inhibition as a therapy for psychiatric disorders.

### ATX inhibition is an effective treatment in a ketamine-induced animal model of schizophrenia

**Fig. 3:**
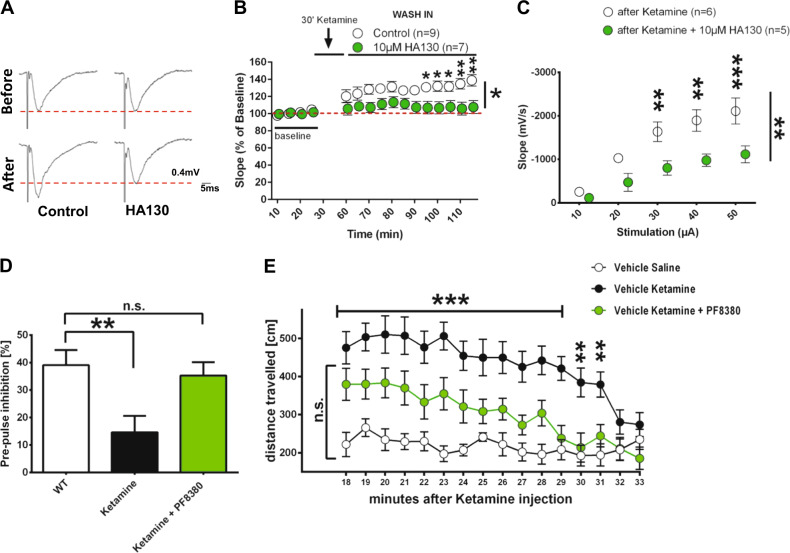
ATX inhibition effectively reduced cortical hyperexcitability, PPI and hyperlocomotion in an animal model for schizophrenia. **a** Representative evoked field potentials of control and 10 µM HA130-treated slices before and after ketamine stimulation. Note the potentiation after ketamine application and the rescued field potentials after HA130 application on ketamine-treated slices. Dotted red lines depict control levels. **b** After an initial depression, ketamine application leads to a significant neuronal potentiation as shown by evoked field potential recordings (1 stimulation every 30 s; for amplitudes see Fig. S4B). However, ATX inhibition by HA130 significantly reduced ketamine potentiation back to baseline levels (*n* = 8 Ketamine and 7 Ketamine + HA130 (10 µM)-treated slices; two-way RM ANOVA and Bonferroni multiple comparison post hoc test). **c** Input/output (I/O) assessment of slopes using increasing stimulus intensities shows that HA130 application significantly decreased I/O-relationship (*n* = 6 Ketamine and 5 ketamine + HA130 (10 µM)-treated slices, two-way RM ANOVA and Bonferroni multiple comparison post hoc test; see also Fig. S4C). **d** Pre-pulse inhibition (PPI) was significantly reduced in ketamine-treated animals but restored to WT levels by ATX inhibition via in vivo application of PF8380. Startle amplitudes were not altered by ketamine application or by ATX inhibition (*n* = 13 control animals, 13 ketamine-treated animals and 16 ketamine + PF8380-treated animals; one-way ANOVA with Bonferroni’s multiple comparisons test; see also Fig. S4D) **e** Ketamine induced significant hyperlocomotion in an open field (OF) setting, whereas in vivo inhibition of ATX via PF8380 administration reduced ketamine-induced hyperlocomotion to WT levels (*n* = 14 control animals, 14 ketamine-treated animals and 15 ketamine + PF8380-treated animals; two-way RM ANOVA and Sidak’s multiple comparison post hoc test). Bars represent mean ± SEM. *p < 0.05, **p < 0.01, ***p < 0.001

As alterations in gamma coherence play a role in psychiatric disorders [30, 32] and ATX inhibition specifically normalized this electrophysiological phenotype, as well as the cortical sensorimotor gating deficit (PPI), we speculated that inhibiting ATX might be an overarching strategy to tackle hyperexcitable cortical states. We therefore analyzed the effect of ATX inhibition in the well-established ketamine model of schizophrenia [16]. Here, short-term application of ketamine induced cortical hyperexcitability as shown by an increase in field potentials (Figs. 3a, b). However, application of HA130, an ATX-inhibitor with short-term action in vitro [5, 22] (Fig. S4A), significantly reduced this increase to baseline levels (Figs. 3a, b). In line with this, analysis of input/output curves, which reflect neuronal excitability, revealed a significant increase after ketamine application, which was significantly reduced following HA130 application (Fig. 3c, Fig. S4C). As ketamine application in vivo results in characteristic behavioral changes related to schizophrenia [16], we analyzed the efficacy of ATX inhibition on reversing these behavioral psychiatric phenotypes. Hereby, we found that ATX inhibition by PF8380 effectively rescued PPI startle responses in this animal model of schizophrenia to wild-type levels (Fig. 3d and Fig S4D) and significantly reduced locomotor hyperactivity, which is a schizophrenia-resembling positive symptom and was characteristically displayed after ketamine application (Fig 3e). In sum, our data suggest that ATX inhibition is a versatile new strategy for tackling cortical hyperexcitability associated with psychiatric disorders.

## Discussion

Bioactive lipid signaling at the synapse has been shown to stimulate glutamatergic transmission, both in the hippocampus [4] and in the somatosensory cortex [6]. This pathway is mediated by LPA acting on G protein-coupled LPA receptors, which induce intracellular signaling [33]. At the synapse, LPA acts on presynaptic LPA2 receptors thereby increasing the presynaptic glutamate release probability [4, 8]. In turn, synaptic LPA appears to be under control of a postsynaptic regulator, PRG-1, which regulates synaptic LPA levels via a cellular uptake mechanism [5]. Thereby postsynaptic PRG-1 regulates LPA stimulation of presynaptic LPA2 receptors, which in turn modulate presynaptic glutamate release probability [4]. The source of synaptic LPA, which has yet to be identified, is a critical point in LPA signaling because LPA is a short-lived molecule that is readily degraded by lipide phosphate phosphatases [34]. Moreover, LPA rapidly integrates into membranes, and is only slightly soluble in aqueous solutions such as the CSF, where it is present in low concentrations [35]. Thus, synaptic LPA signaling requires an—as of yet unclear—local LPA source close to its point of action at the synapse. LPA is synthesized in the extracellular space by the lysoPLD ATX, which hydrolyzes LPC to LPA [36, 37]. Although in the blood LPC is abundant, it is actively transported across the blood–brain barrier and is present in the CSF only in minimal concentrations [35]. The low availability of the LPA precursor LPC in the brain together with the rapid degradation of LPA makes the source of synaptic LPA a critical factor for synaptic LPA signaling.

### ATX expression in PAPs is a characteristic feature of excitatory cortical synapses and fuels glutamatergic synaptic lipid signaling

Electron microscopic analyzes revealed ATX-expressing PAPs, which were found adjacent to asymmetric, excitatory cortical synapses but not to inhibitory synapses. Together with previous reports showing that LPA signaling at glutamatergic synapses is mediated by the presynaptic LPA2 receptor and is regulated by the postsynaptic LPA-interacting molecule PRG-1 [4, 5], our data describe a novel molecular mechanism regulating glutamatergic transmission at the tripartite glutamatergic synapse. This synaptic ATX localization is optimally suited to provide fast local LPA levels at a timescale necessary for modulation of glutamatergic transmission. Importantly, astrocytic ATX release and increased LPA production were dynamically regulated in a glutamate-dependent manner, which involved astrocytic AMPA and NMDA receptors. These data suggest that the ATX/LPA/LPA2 signaling axis might act as a feed-forward enhancer following neuronal activity: at the synapse, increased glutamate levels following neuronal firing would result via astrocytic stimulation in increased synaptic LPA levels, which in turn would enhance the presynaptic glutamate release probabilities. Indeed, these effects were observed in PRG-1^-/-^ animals where the PRG-1-dependent regulation is missing [4, 6, 8]. The specific effect of synaptic ATX was further corroborated by cell type-specific genetic deletion of ATX in astrocytes, which rescued neuronal hyperexcitability in PRG-1^-/-^ animals to wild-type levels. Moreover, ATX inhibition rescued disturbed presynaptic plasticity in PRG-1^-/-^ mice confirming the presynaptic action of synaptic LPA. In sum, our data suggest that ATX is a key molecule within a stimulatory feed-forward mechanism of excitatory transmission and is in line with recent reports suggesting that astrocytes are a novel regulatory element of excitatory synaptic function acting via lipid-mediated pathways [38].

### Inhibition of ATX as a new strategy for treating psychiatric disorders

Shifts in the (E/I) balance of cortical networks toward a higher excitability leading to pathological cortical information processing have been suggested to contribute to the pathophysiology of psychiatric disorders like schizophrenia and are considered biomarkers/endophenotypes for this disorder [3, 10, 12, 39–41]. Using different genetically modified mouse lines where alterations in synaptic lipid signaling resulted in a cortical network hyperexcitability (PRG-1^-/-^ described in Trimbuch et al. [4] and PRG-1^R346T^, expressing a human PRG-1 mutation described in Vogt et al. [5]) we found a significant augmentation of gamma coherence, a pathological PPI and hyperlocomotion. These findings are of specific importance as these symptoms have been implicated in psychiatric disorders like schizophrenia [30, 32, 42, 43], and were normalized to wild-type levels by ATX inhibition. Moreover, they provided a strong argument to consider ATX inhibition as a general therapeutic approach to treat cortical hyperexcitable states. We therefore analyzed the effect of ATX inhibition in a well-described animal model for schizophrenia, where application of ketamine increased cortical excitability as recently described in mice and related to findings in human schizophrenia patients [16]. Here, ATX inhibition was effective in normalizing both neuronal hyperexcitation, as well as pathological behaviors including PPI and hyperlocomotion in this animal model for schizophrenia demonstrating the suitability of targeting synaptic bioactive lipid signaling as a potential therapy for psychiatric disorders.

ATX inhibition by small inhibiting molecules like PF8380 is a rapidly evolving treatment strategy, which was used for chronic inflammation and cancer with a potential therapeutic benefit [44], as well as for treating air-pouch inflammation [23], collagen-induced arthritis [45], bleomycin-induced pulmonary inflammation and fibrosis [46], allergen-induced asthma [47], or metastasis of melanoma and breast cancer cells [45–52]. Moreover, our data showing that ATX inhibition under physiological conditions does not alter cortical electrophysiology and does not affect animal behavior suggests that ATX inhibition is a versatile strategy for treating cortical hyperexcitatory states present in psychiatric disorders while not affecting individuals with physiological brain states.

## Electronic supplementary material

Supplemental Fig S1-4
