# Supplementary material for: Synaptic phospholipids as a new target for cortical hyperexcitability and E/I balance in psychiatric disorders
Source: Mol Psychiatry. 2018 May 9;23(8):1699–710. doi: 10.1038/s41380-018-0053-1 (PMC6153268; doi:10.1038/s41380-018-0053-1)
Supplement: Supplementary file 1 — Supplemental Fig S1-4 [file 41380_2018_53_MOESM1_ESM.pdf]

Supplemental Information

Synaptic bioactive phospholipid signaling:

A new target for treating psychiatric disorders

Running title: Bioactive lipids in psychiatric disorders

Carine Thalman*, Guilherme Horta*, Lianyong Qiao*, Heiko Endle, Irmgard Tegeder, Hong Cheng, Gregor Laube, Torfi Sigrudsson, Maria Jelena Hauser, Stefan Tenzer, Ute Distler, Junken Aoki, Gerd Geisslinger, Jochen Roeper, Sergei Kirischuk, Heiko J. Luhmann, Konstantin Radyushkin, Robert Nitsch[#] and Johannes Vogt[#]

Supplemental Figure S1. related to Fig. 1

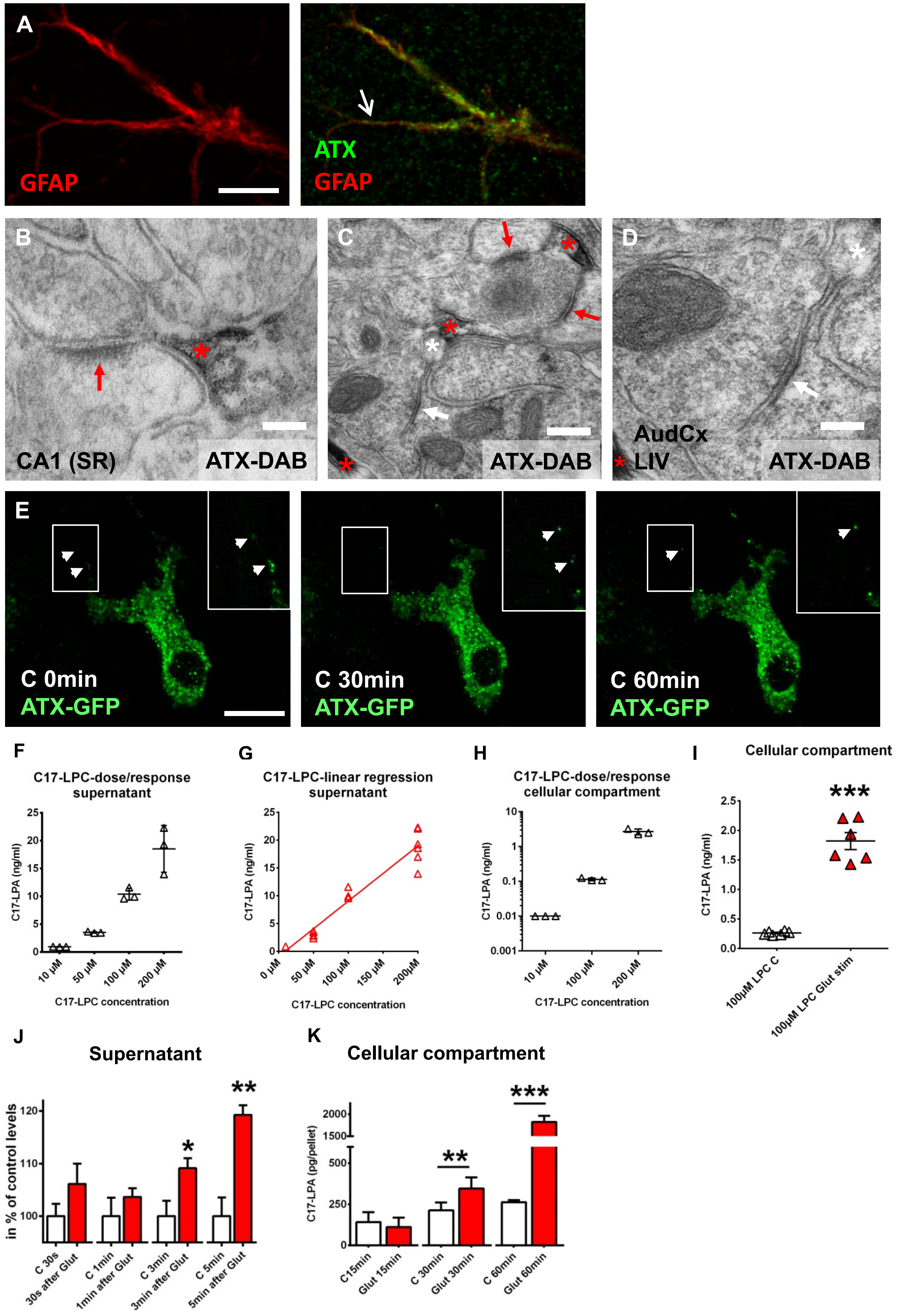

Supplemental Figure S1. related to Fig. 1:

ATX is expressed in astrocytic processes and is dynamically regulated by glutamate.

A. High-sensitivity immunofluorescence detection of autotaxin revealed ATX-positive punctae along GFAP-positive astrocytic processes Scale bar: 10 µm.

B. Electron microscopic picture of the hippocampal CA1-stratum radiatum (SR) displaying ATX-expression in a perisynaptic astrocytic process at a glutamatergic synapse (red arrow) depicted by DAB-precipitation. Remarkably, ATX-immunosignal in the astrocytic process shows an expression gradient towards the synapse and is prominently found at the membranes close to the synaptic cleft (red asterisk). Scale bar: 200nm.

C. Electron microscopic image of the layer4 auditory cortex reveals excitatory synapses (red arrow) surrounded by ATX-expressing perisynaptic processes (red stars). In contrast a perisynaptic astrocytic process (white star) in close vicinity to an inhibitory synapse (white arrow) does not express ATX. Note the ATX-expression in a vessel at the lower left edge (red asterisk). Scale bar: 300nm.

D. Higher magnification of the inhibitory synapse shown in C. Scale bar: 100nm.

E. Live-imaging of transfected ATX-GFP astrocytes kept under control conditions (media change was performed to avoid bias induced by shear stress from the media change itself) and imaged at the same time points as astrocytes after glutamate stimulation. Inset (right) depicts a higher magnification of the boxed area including a astrocytic process. Arrows in inserts point to sparse ATX-GFP vesicles in this astrocytic process imaged at the indicated time points. Scale bar: 10 µm.

F. C17-LPA concentrations in the supernatant as a direct measure of astrocytic ATX activity were measured by mass spectrometry after 1h incubation with different concentrations of the unnatural LPA-precursor C17-LPC.

G. Linear regression on data pooled from multiple experiments suggests a linear correlation of C17-LPA concentration in the supernatant to the C17-LPC in the media after 1h incubation time.

H. C17-LPA levels in the astrocytic compartment after 1h incubation with different C17-LPA concentrations.

I. C17-LPA concentration in the cellular compartment after 1h was significantly increased upon 15 min of 500 µM glutamate stimulation when compared to non-stimulated controls (media change only; n = 6 per group, paired t-test).

J. Upon addition of the unnatural LPA-precursor C17-LPC, C17-LPA synthesis, a direct correlate of ATX activity, was detected at significantly higher levels in the supernatant of glutamate-stimulated astrocytes than in supernatant from control (C), non-stimulated astrocytic cultures as shown by mass spectrometric analyses (n = 6 experiments per group, one-tailed paired t-test).

K. C17-LPA concentrations over time after incubation with 100 µM LPC after 15, 30 or 60 min. (n = 9 control (C) 15 min, n = 8 Glut 15 min, n = 6 for all other conditions, Wilcoxon matched-pairs signed rank test for 15 min and paired t-test for 30 min and 60 min).

Bars represent mean ± SEM. *$p < 0.05$, **$p < 0.01$, ***$p < 0.001$.

Supplemental Figure S2. related to Fig. 2

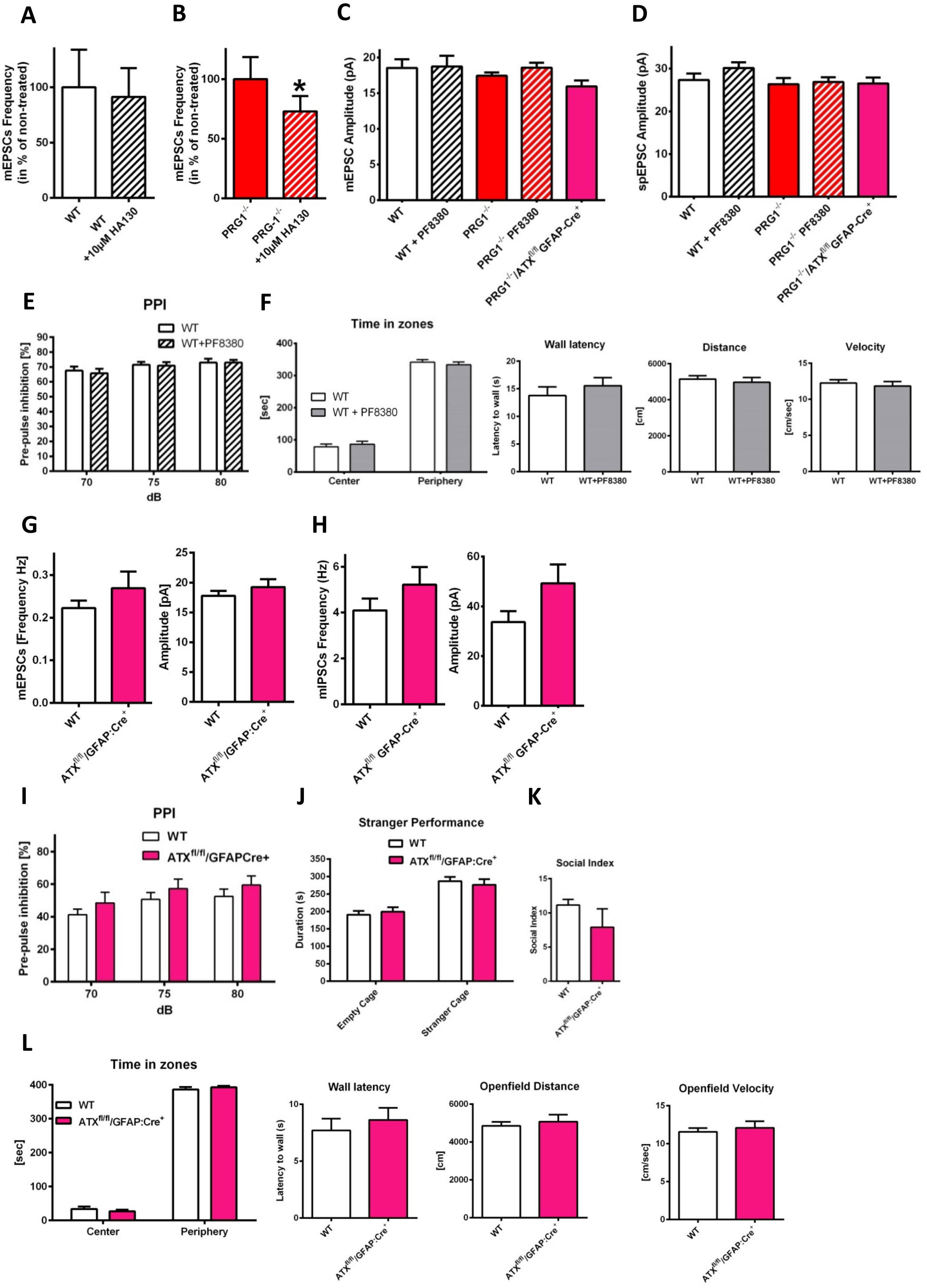

Supplemental Figure S2. related to Fig. 2:

ATX-inhibition does not alter mEPSCs, mIPSCs, cortical sensory information processing and cortical behavior under physiological conditions.

A. mEPSC measurements in WT neurons before and after ATX-inhibition performed in the same neuron (N=7 neurons, two-tailed Mann-Withney-test).

B. mEPSC measurements in PRG-1$^{-/-}$ neurons before and after ATX-inhibition performed in the same neuron (N=8 neurons, two-tailed t-test). While ATX-inhibition did not change mEPSC frequency in WT neurons, mEPSCs in PRG-1$^{-/-}$ neurons significantly decreased upon ATX-inhibition.

C,D. Amplitudes of (C) miniature excitatory postsynaptic currents (mEPSCs) and (D) spontaneous excitatory postsynaptic currents (spEPSCs) as analyzed in Fig. 2A and C. No difference was observed between the different conditions (one-way ANOVA).

E. Pre-pulse inhibition (PPI) in wild type (WT) and in PF8380-treated WT animals (n = 14 WT and 16 WT + PF8380; two-way RM ANOVA).

F. Open field (OF) behavior in WT and PF8380-treated WT animals (n = 14 WT + vehicle and 16 WT + PF8380; for wall latency n = 10 WT + vehicle and 14 WT + PF8380; two-tailed t-test)

G. mEPSC frequency and amplitudes with astrocyte-specific ATX-deletion (n=11 WT neurons and 12 ATX$^{fl/fl}$/GFAP-Cre^{+} neurons, two-tailed t-test).

H. mIPSC frequency and amplitudes with astrocyte-specific ATX-deletion (n=8 WT neurons and 10 ATX$^{fl/fl}$/GFAP-Cre^{+} neurons, two-tailed t-test).

I. Pre-pulse inhibition (PPI) in animals with cell-type specific astrocytic ATX-deletion (ATX$^{fl/fl}$/GFAP Cre^{+}) and in their wild type litters (WT/ATX$^{fl/fl}$/GFAP Cre^{-}) (n = 12 WT and 12 ATX$^{fl/fl}$/GFAP Cre^{+}; two-way RM ANOVA).

J,K. Stranger performance and social index in WT(ATX$^{fl/fl}$/GFAP Cre^{-}) and ATX$^{fl/fl}$/GFAP Cre^{+} litters (n = 12 WT and 12 ATX$^{fl/fl}$/GFAP Cre^{+}; two-tailed t-test).

L. OF test of WT(ATX$^{fl/fl}$/GFAP Cre^{-}) and ATX$^{fl/fl}$/GFAP Cre^{+} (cell-type specific ATX-deficient) litters (two-tailed t-test).

Bars represent mean ± SEM. *$p < 0.05$

Supplemental Figure S3. related to Fig. 2

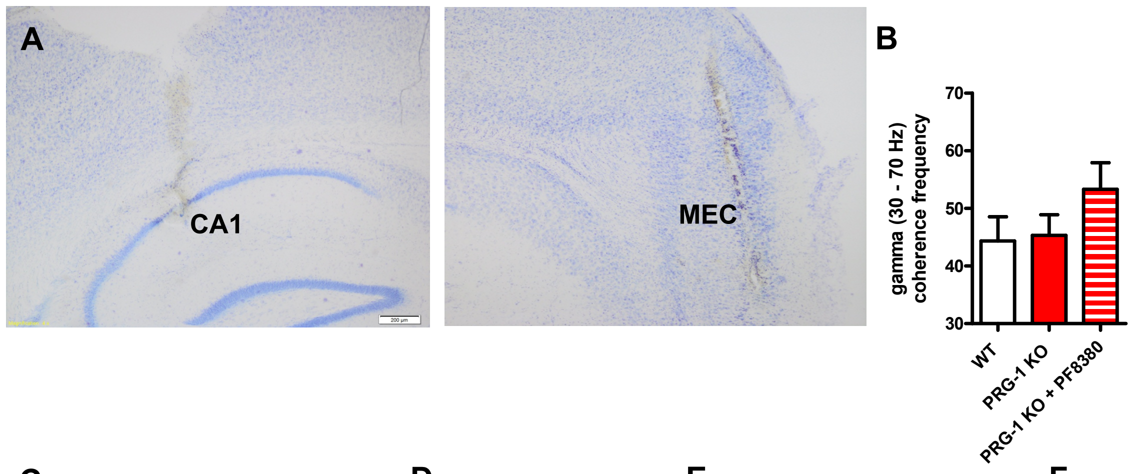

C

Concentration of PF 8380 *in-vivo* assessed by LC-MS/MS
(3h after i.p. injection of 30 mg/KG/BW)

PF8380	Value	c(pmol/µl)
Serum 1	2.88 E+007	15.9
Serum 2	5.43 E+006	2.97
CSF1	6.58 E+06	0.36
CSF2	7.57 E+06	0.42

IC50 = 101 nM (0.1 pMol/µl)

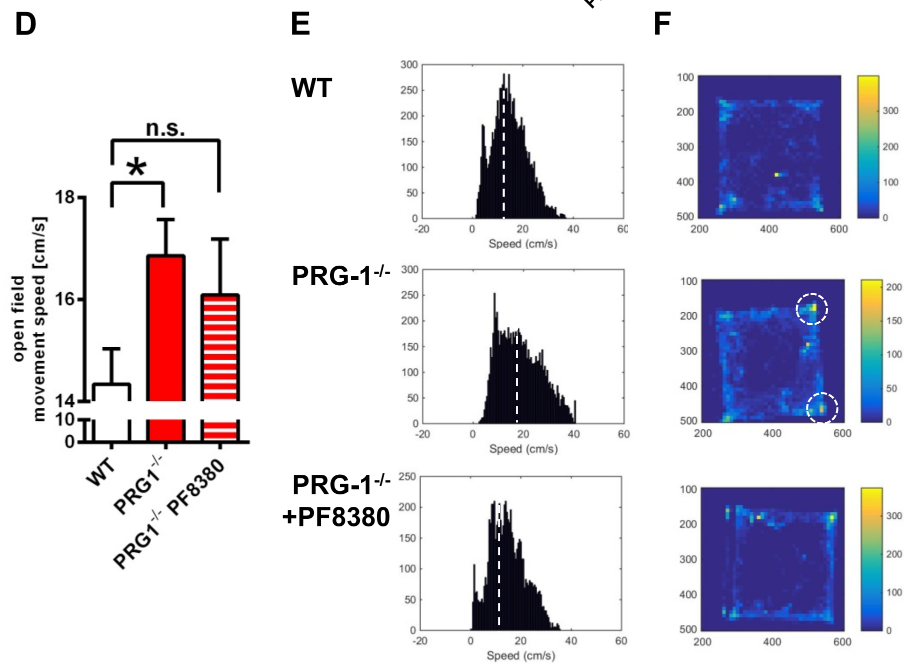

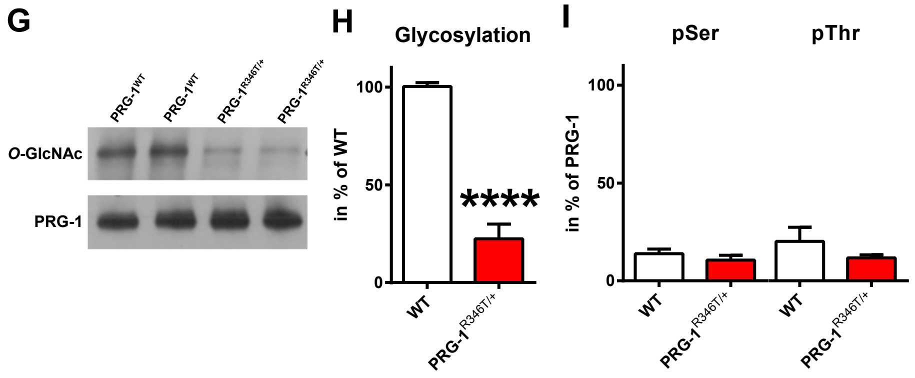

Supplemental Figure S3. related to Fig. 2:

In vivo recordings in interconnected cortico-hippocampal regions in freely moving WT and PRG-1$^{-/-}$ mice and characteristics of a human PRG-1 SNP-expressing mouse line (PRG-1^{R346T})

A. Nissl-stained brain slices depict the electrode position in the hippocampal CA1 region and in layer II/III of the medial entorhinal cortex (MEC). Scale bar: 200 µm.

B. The gamma coherence frequency peak was not significantly altered between wild type (WT) and PRG-1$^{-/-}$ or PF8380-treated PRG-1$^{-/-}$ animals.

C. In vivo application of PF8380 in therapeutic concentrations of 30 mg/kg body weight i.p. resulted in robust concentrations of PF8380 in the blood serum and effective levels in the cerebrospinal fluid (CSF) which were up to 4 times higher than IC50.

D. Analysis of mean speed movement revealed a significant increase in PRG-1$^{-/-}$ mice, while PF8380-treatement rescued hypermotility in PRG-1$^{-/-}$ to WT levels (n= 15 WT, 10 PRG-1$^{-/-}$ and 6 PRG-1$^{-/-}$ + PF8380 treatment, one-way ANOVA with Bonferroni post hoc).

E. Representative graphic display of the speed movement of WT litters, PRG-1$^{-/-}$ and PF-8083 treated PRG-1$^{-/-}$ mice revealed clear differences in speed movement. Average speed in these animals is indicated by dotted white line.

F. Heat-map shows localization of the mice during OF behavior. Here, PRG-1$^{-/-}$ mice displayed a preference for corners (shown by dotted circles), which is in line with recent published data (Schneider et al., 2017).

G. *O*-Glycosylation of PRG-1 IP-product (PRG-1 input shown below) in WT and in PRG-1$^{R346T/+}$ brains as assessed by Western blot. *O*-Glycosylation of PRG-1 shows decreased levels in PRG-1$^{R346T/+}$ brains and is in line with molecular findings in heterologous systems and in-vitro experiments , which showed that PRG-1$^{R346T/+}$ is a loss-of-function mutation (Vogt et al. 2016).

H. Quantitative analysis of Western blot grey values normalized to PRG-1 input confirms the significant decrease in *O*-linked PRG-1 glycosylation in PRG-1$^{R346T/+}$ brains, which is in line with previously published data in hetereolgous systems (n=7 WT and 7 PRG-1$^{R346T/+}$ brains, Vogt et al., 2016).

I. Quantitative analysis of Western blot grey values show low Ser and Thr phosphorylation of PRG-1 IP-product in WT and PRG-1$^{R346T/+}$ brains, which corresponds to previous findings showing low pS347 levels as measured by mass spectrometry in hetereologous systems (n=6 WT and 6 PRG-1$^{R346T/+}$ brains, Vogt et al., 2016).

Bars represent mean ± SEM. *p < 0.05, ****p<0.0001.

Supplemental Figure S4. related to Fig. 3

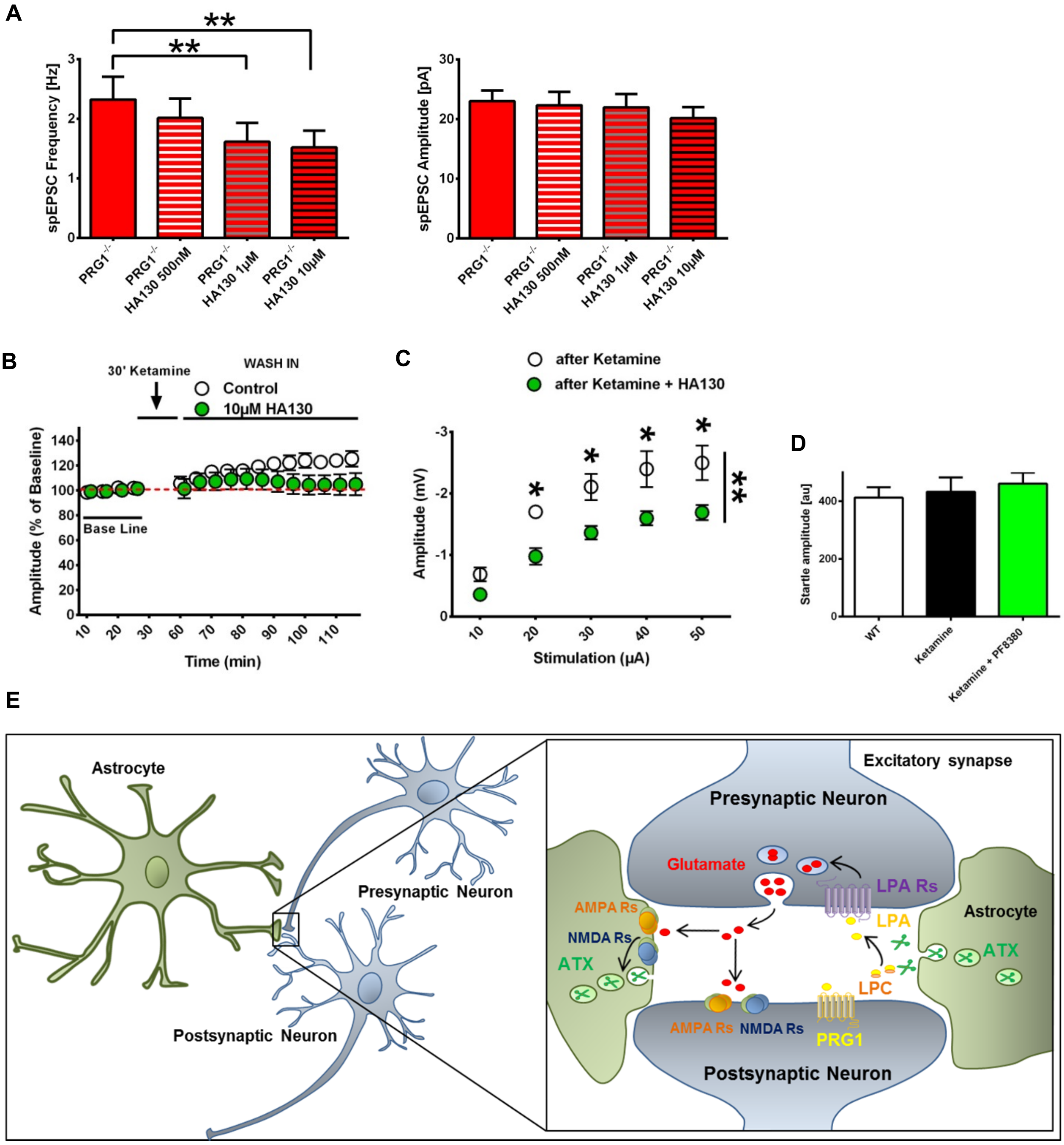

Supplemental Figure S4. related to Fig. 3:

ATX-inhibition reverts hyperexcitatory cortical states.

A. Increasing concentrations of HA130, which blocks the LPA synthesis molecule autotaxin (ATX), significantly reduced spontaneous postsynaptic currents (right), which are the sum of the overall excitatory and inhibitory input, on single neuronal level while not affecting their amplitude (n = 10 PRG-1$^{-/-}$ neurons; one-way RM ANOVA with Bonferroni post hoc).
B. Amplitudes of field potential recordings after ketamine application and subsequent HA130 treatment (n = 8 ketamine and 7 ketamine + HA130 (10 µM)-treated slices; two-way RM ANOVA and Bonferroni multiple comparison post hoc test).
C. Amplitudes of input/output measurements were significantly decreased after HA130 treatment (n = 6 ketamine and 5 ketamine + HA130 (10 µM)-treated slices; two-way RM ANOVA and Bonferroni multiple comparison post hoc test).
D. Startle amplitudes of wild type (WT) and ketamine-treated mice as shown in Fig 3D (n = 13 control animals, 13 ketamine-treated animals and 16 ketamine + PF8380-treated animals; one-way ANOVA with Bonferroni`s multiple comparisons test).
E. Schematic representation of the analyzed phospholipid regulated glutamatergic transmission at the tripartite glutamatergic synapse, which consists of the perisynaptic astrocytic processes (PAPs), ensheathing the synapse, and of the pre- and postsynaptic compartments. Briefly, our data point to the fact that presynaptic glutamate acts via astrocytic AMPA, NMDA and metabotropic glutamate receptors leading to an increased astrocytic ATX transport along astrocytic processes which is released at the synapse. Here, ATX converts LPC to LPA, which acts via presynaptic LPA2-receptors thereby stimulating presynaptic glutamate release.
Bars represent mean ± SEM. *p < 0.05, **p < 0.01.
